# An association between decreasing incidence of invasive non-typhoidal salmonellosis and increased use of antiretroviral therapy, Gauteng Province, South Africa, 2003–2013

**DOI:** 10.1371/journal.pone.0173091

**Published:** 2017-03-06

**Authors:** Karen H. Keddy, Simbarashe Takuva, Alfred Musekiwa, Adrian J. Puren, Arvinda Sooka, Alan Karstaedt, Keith P. Klugman, Frederick J. Angulo

**Affiliations:** 1 Centre for Enteric Diseases, National Institute for Communicable Diseases, National Health Laboratory Service, Johannesburg, South Africa; 2 Faculty of Health Sciences, University of the Witwatersrand, Johannesburg, South Africa; 3 CARTA Africa, Nairobi, Kenya; 4 Centre for HIV, National Institute for Communicable Diseases, National Health Laboratory Service, Johannesburg, South Africa; 5 International Emerging Infections Program, South Africa Global Disease Detection Centre, Centers for Disease Control and Prevention, Pretoria, South Africa; 6 Department of Medicine, Chris Hani Baragwanath Hospital, Johannesburg, South Africa; 7 Bill and Melinda Gates Foundation, Seattle, WA, United States of America; 8 Division of Global Health Protection, Center for Global Health, Centers for Disease Control and Prevention, Atlanta, GA, United States of America; Vanderbilt University, UNITED STATES

## Abstract

**Background:**

HIV-infected persons are at increased risk of opportunistic infections, including invasive nontyphoidal *Salmonella* (iNTS) infections; antiretroviral therapy (ART) reduces this risk. We explored changing iNTS incidence associated with increasing ART availability in South Africa.

**Methods:**

Laboratory-based surveillance for iNTS was conducted in Gauteng Province, South Africa, with verification using the National Health Laboratory Service’s Central Data Warehouse (CDW), between 2003 and 2013. Isolates were serotyped at the Centre for Enteric Diseases. CDW data on patient numbers obtaining HIV viral load measurements provided estimates of numbers of HIV-infected patients receiving ART. A Poisson regression model was used to measure the changing incidence of iNTS infection from 2003 to 2013. The correlation between the incidence of iNTS and ART use from 2004 to 2013 was determined using Pearson’s correlation coefficient.

**Results:**

From 2003–2013, the incidence of iNTS per 100,000 population per year decreased from 5.0 to 2.2 (p < .001). From 2004 to 2013, the incidence per 100,000 population of HIV viral load testing increased from 75.2 to 3,620.3 (p < .001). The most common serotypes causing invasive disease were *Salmonella enterica* serovar Typhimurium (*Salmonella* Typhimurium), and *Salmonella* Enteritidis: 2,469 (55.4%) and 1,156 (25.9%) of 4,459 isolates serotyped, respectively. A strong negative correlation was observed between decreasing iNTS incidence and increasing ART use from 2004 to 2013 (r = -0.94, p < .001). Similarly, decreasing incidence of invasive *Salmonella* Typhimurium infection correlated with increasing ART use (r = -0.93, p < .001). Incidence of invasive *Salmonella* Enteritidis infection increased, however (r = 0.95, p < .001). Between 2003 and 2004, fewer adult men than women presented with iNTS (male-to-female rate ratio 0.73 and 0.89, respectively). This was reversed from 2005 through 2013 (ranging from 1.07 in 2005 to 1.44 in 2013). Adult men accessed ART less (male-to-female rate ratio ranging from 0.61 [2004] to 0.67 [2013]).

**Conclusions:**

The incidence of iNTS infections including *Salmonella* Typhimurium decreased significantly in Gauteng Province in association with increased ART utilization. Adult men accessed ART programs less than women, translating into increasing iNTS incidence in this group. Monitoring iNTS incidence may assist in monitoring the ART program. Increasing incidence of invasive *Salmonella* Enteritidis infections needs further elucidation.

## Introduction

Despite the introduction and rapid uptake of antiretroviral therapy (ART) in South Africa in 2004 [1], South Africa has among the highest prevalence of HIV infection in the world [2]. In 2013, the estimated national seroprevalence of HIV infection in South Africa was 15% in adults aged 15–49 years [3], with an estimated 1000 new infections daily [4]. High prevalence of HIV infection in South Africa places a tremendous burden on the health systems within the country, due largely to opportunistic infections, including invasive nontyphoidal *Salmonella* (iNTS) infection. In parts of South Africa where ART coverage is high, declining HIV transmission rates and improvements in health-related quality of life have been noted [5]. Previous researchers have attempted to measure the impact of increased ART use among HIV-infected persons on the incidence of opportunistic infections in South Africa, with mixed results. Nunes *et al* showed increased ART use among HIV-infected persons was associated with declining morbidity and mortality in HIV-infected children due to invasive pneumococcal disease [6], but were unable to replicate these findings in HIV-infected adults [7]. Nanoo *et al* described a temporal decrease in the incidence of microbiologically confirmed tuberculosis with increase antiretroviral use among HIV-infected individuals in South Africa [8], based on estimates of ART use, derived by the Actuarial Society of South Africa (ASSA) [3].

The impact of increased ART use among HIV-infected persons on the incidence of iNTS in South Africa has not been defined, despite the well-described association of *Salmonella enterica* serotype Typhimurium (*Salmonella* Typhimurium) ST313 with iNTS among HIV-infected persons in Africa [9–12]. At the Queen Elizabeth Central Hospital in Malawi, following the introduction of ART, a 36% reduction in iNTS incidence in adults was described [13]: however, it was unclear how much of the reduction in iNTS incidence in children in Malawi could be ascribed to increased ART use since there were also improvements in nutrition status and declines in prevalence of malaria during this period [14]. Researchers in other African countries have also recently described declines in iNTS incidence, but have not specifically related this decline to increased ART use [15,16]. Concerns have also been raised that adult men may not be accessing ART programs: Bor *et al* reported HIV-related mortality in a rural area of South Africa declined significantly more in women than men (p = .05) [17]. This study was undertaken to describe associations between iNTS incidence and ART use among HIV-infected persons in Gauteng Province, South Africa, a malaria-free province with an urbanised population with good health care access [4], low prevalence of malnutrition, good access to safe water and sanitation and an HIV-seroprevalence of 11% [3].

## Methods

### Invasive non-typhoidal *Salmonella*

Between 2003 and 2013, the Centre for Enteric Diseases (CED) undertook active laboratory-based surveillance for invasive non-typhoidal *Salmonella* (iNTS) at clinical diagnostic laboratories in Gauteng Province (S1 Dataset). Laboratories were requested to submit all *Salmonella* isolated from normally sterile sites to CED for further characterization, including serotyping. We defined iNTS infection as the isolation of non-typhoidal *Salmonella* from a normally sterile body site. Data at the Central Data Warehouse (CDW) of the National Health Laboratory System (NHLS) were reviewed to confirm reporting of all iNTS infections: CDW is a repository for all public sector laboratory results in South Africa (representing over 80% of all facilities, >43 million people) which includes all microbiology and HIV-related laboratory tests conducted across public health laboratories. We recorded data on age and sex for all patients presenting with iNTS infection. Data were de-duplicated: i.e. each episode of iNTS was counted as a single event, even if multiple isolates were obtained from a patient for each admission. If a patient presented 21 days or more with a second iNTS infection, this was counted as a new episode. Data were not anonymised prior to analysis. All *Salmonella* isolates received were serotyped following CED standard operating procedures (Mast Group, Merseyside, UK; BioRad, Marnes-la-Coquette, France; Remel, Kent, UK; Statens Serum Institute, Copenhagen, Denmark). Data were recorded in an Access 2007 database (Microsoft, Seattle, USA).

### Antiretroviral use among HIV-infected persons

Since the availability of ART in 2004, routine management of HIV-infected persons in South Africa includes ART and measurement of HIV viral load at least annually; most patients on ART in South Africa have viral load testing performed at a NHLS laboratory. We therefore used CDW’s data on HIV viral load measurement tests to estimate the number of persons in Gauteng Province using ART from 2004 to 2013. Viral load data were de-duplicated by removing repeat viral load tests performed on the same patient in the same calendar year (S1 Table). As a sensitivity analysis, we compared our estimate of the number of HIV-infected persons ≥15 years of age on ART to limited unpublished data from National Department of Health (NDoH) reports [18]. These data were anonymised prior to analysis.

### Statistical analysis

To derive estimates of the incidence of iNTS and estimates of the incidence of the number of persons on ART, population denominators were derived from mid-year data estimates data published annually by the national Department of Statistics (www.statssa.gov.za). Incidence was also estimated for the following age groups: <5 years; 5–14 years; 15–24 years; 25–49 years and ≥50 years.

A Poisson regression model was used to measure the change in incidence of iNTS infection from 2003 to 2013, and change in incidence of ART use from 2004 to 2013. We additionally determined the correlation between the incidence of iNTS and ART use from 2004 to 2013 using Pearson’s correlation coefficient. Analyses were performed using Stata version 13 (StataCorp Limited, College Station, TX, USA).

## Results

### Invasive non-typhoidal *Salmonella*

**Fig 1:**
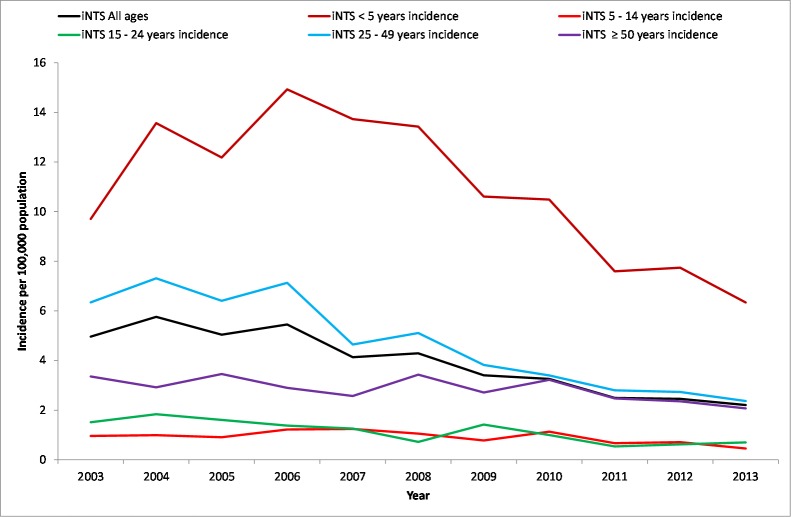
Incidence of invasive *Salmonella* per 100,000 population per year in Gauteng Province, South Africa, by age group, 2003–2013. (Test for trend: All ages, incidence rate ratio (IRR) = 0.91, 95% CI = 0.90–0.92, p < .001; <5 years, IRR = 0.95, 95% CI = 0.93–0.96, p < .001; 5–14 years, IRR = 0.95, 95% CI = 0.91–0.99, p = .03; 15–24 years, IRR = 0.90, 95% CI = 0.87–0.94, p < .001; 25–49 years, IRR = 0.89, 95% CI = 0.88–0.90, p < .001; ≥50 years, IRR = 0.96, 95% CI = 0.94–0.99, p = .007).

From January 2003 –December 2013, we identified 4,886 cases of iNTS from Gauteng Province, South Africa. Sex was recorded for 4,728 (96.8%) patients: 2,478 (52.4%) were male. Ages were available for 4,661 (95.4%) patients, ranging from 0 days (newborn) to 93 years, with a median of 32 years. Incidence of iNTS (cases per 100,000 population) per year increased from 5.0 in 2003 to 5.8 in 2004, after which there was a steady decrease to 2.2 in 2013 (Table 1). The highest incidence of iNTS was in children <5 years of age; the lowest incidence was in children aged from 5 to 14 years (Fig 1). Prevalence of HIV infection decreased from 3,680.3 to 3,041.4 per 100,000 in children aged <5 years, but increased in children aged 5to 14 years from 480.1 to 2,877.1 per 100,000 between 2003 and 2013 (S1 Table). Among adults, HIV prevalence decreased in those aged 15–24 years (from 8,332.3 to 4,641.9 per 100,000). HIV prevalence stabilised in those aged 25 to 49 years (from 16,643.2 and16,917.7 per 100,000) and increased in those aged 50 years and older (from 2,919.0 to 5,722.8 per 100,000) respectively over the period (S1 Table).

**Table 1:** Population and number (incidence per 100,000 population) of invasive non-typhoidal *Salmonella* (iNTS) cases per year, Gauteng Province, South Africa, 2003–2013.

Year	Population*	Number of iNTS cases (iNTS incidence)	
2003	10,273,446	510	(5.0)
2004	10,500,732	605	(5.8)
2005	10,730,594	541	(5.0)
2006	10,964,701	598	(5.5)
2007	11,202,290	463	(4.1)
2008	11,445,709	491	(4.3)
2009	11,693,933	398	(3.4)
2010	11,946,060	389	(3.3)
2011	12,202,306	304	(2.5)
2012	12,463,886	306	(2.5)
2013	12,728,438	281	(2.2)

*www.statssa.gov.za.

Serotyping was completed on 4,459 (91.2%) of isolates: the most common serotypes were *Salmonella enterica* serovar Typhimurium (*Salmonella* Typhimurium) 2,469 (55.4%) and *Salmonella* Enteritidis, 1,156 (25.9%).

The incidence of iNTS decreased from 2003 to 2013 (incidence rate ratio [IRR] = 0.91, 95% confidence interval [CI] = 0.90–0.92, p < .001) (Fig 1; S2 Table). By serotype, invasive *Salmonella* Typhimurium infections also decreased between 2003 and 2013 (IRR = 0.79, 95% CI = 0.78–0.81, p < .001) (S2 Table). Invasive *Salmonella* Enteritidis infections, however increased over the same period (IRR = 1.14, 95% CI 1.12–1.22, p < .001) (S2 Table).

### ART use

After removing repeat viral load tests on the same patient during the same calendar year, 1,940,203 viral load tests were done in Gauteng between 2004 and 2013. Of these, 677,246 (35.3%) were done on male patients (19,511 had no gender stated) and 174,477 (9.0%) were done on children <15 years of age (S1 Table). Annually, the number of viral load tests increased as follows: 7,849 tests in 2004 (75.2 per 100,000 population); 2005: 35,059 tests (326.7 per 100,000 population); 2006: 79,671 tests (726.6 per 100,000 population); 2007: 118,550 tests (1058.3 per 100,000 population); 2008: 176,950 tests (1546.0 per 100,000 population); 2009: 209,445 tests (1791.1 per 100,000 population); 2010: 220,217 tests (1843.4 per 100,000 population); 2011: 271,179 tests (2222.4 per 100,000 population); 2012: 360,432 tests (2,891.8 per 100,000 population); 2013: 460,806 tests (3,620.3 per 100,000 population).

### Association between incidence of iNTS and incidence of ART use

**Fig 2:**
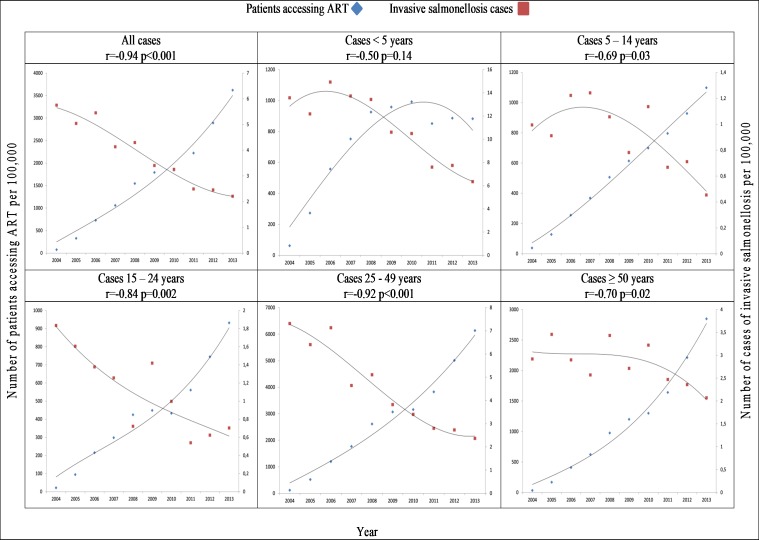
Comparison of incidence of invasive *Salmonella* (iNTS) per 100,000 population per year by age range and incidence of number of patients accessing antiretroviral therapy (ART) per 100,000 population by age range, Gauteng Province, South Africa, 2004–2013.

**Fig 3:**
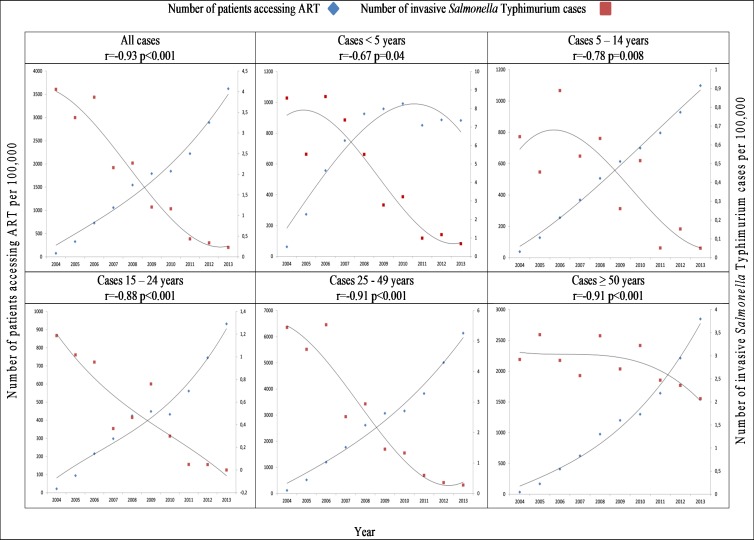
Comparison of incidence of invasive *Salmonella* Typhimurium per 100,000 population per year by age range, and incidence of number of patients accessing antiretroviral therapy (ART) per 100,000 population by age range, Gauteng Province, South Africa, 2004–2013.

**Fig 4:**
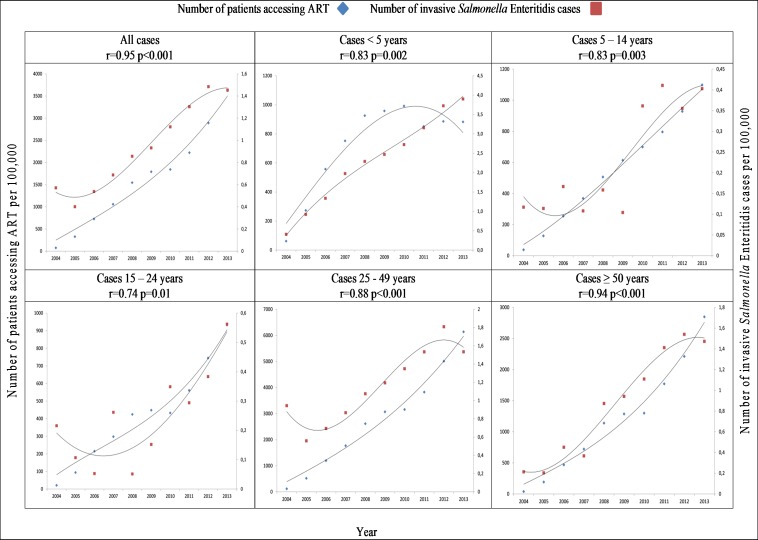
Comparison of incidence of invasive *Salmonella* Enteritidis per 100,000 population per year by age range, and incidence of number of patients accessing antiretroviral therapy (ART) per 100,000 population by age range, Gauteng Province, South Africa, 2004–2013.

There was a correlation between increased use of ART and decreased incidence of iNTS infection per 100,000 population per year from 2004 to 2013 in all age groups (r = -0.94, p < .001). This correlation was related to those patients ≥5 years of age: (5–14 years, r = -0.69, p = .03; 15–24 years, r = -0.84, p = .002; 25–49 years, r = -0.92, p < .001; ≥50 years, r = -0.70, p = .02) (Fig 2; S3 Table). Specifically analysing invasive disease due to *Salmonella* Typhimurium, there was a significant decrease in incidence which correlated with the increased use of ART from 2004 to 2013 (r = -0.93, p < .001) (Fig 3; S4 Table). This correlation was not observed with invasive disease due to *Salmonella* Enteritidis, which increased significantly over the period (r = 0.94, p < .001) (Fig 4; S5 Table).

Comparing rates of iNTS infection against numbers of HIV-infected adult men versus those in HIV-infected adult women, between 2003 and 2004, fewer men presented with iNTS but this was reversed from 2005 through 2013. Regarding access to antiretrovirals, adult men in Gauteng Province accessed ART consistently less than adult women, ranging from a rate ratio of 0.61 in 2004 to 0.67 in 2013 (Table 2).

**Table 2:** Comparison of number of HIV-infected adult men and women (> 15 years), invasive nontyphoidal *Salmonella* incidence rates in adult men and women and adult men and women accessing antiretroviral therapy (ART) in Gauteng Province, South Africa, 2004–2013.

Year	Number of HIV-infected patients [3]		Number of invasive salmonellosis cases				Male-to-female rate ratio	95% Confidence interval	*P*	Patients accessing ART				Male-to-female rate ratio	95% Confidence interval	*P*
	Male	Female	Male	iNTS/ 100,000HIV-infected	Female	iNTS/ 100,000HIV-infected				Male	ART/ 1,000 HIV-infected	Female	ART/ 1,000 HIV-infected			
2003	457,906	475,113	137	29.9	195	41.0	0.73	0.58–0.91	0.002	-	-	-	-	-	-	-
2004	478,250	503,872	185	38.7	218	43.3	0.89	0.73–1.09	0.1	2,282	4.77	3,921	7.78	0.61	0.58–0.64	<0.001
2005	490,114	526,817	187	38.1	187	35.5	1.07	0.87–1.32	0.2	9,924	20.24	18,135	34.42	0.59	0.57–0.60	<0.001
2006	495,452	546,123	221	44.6	181	33.1	1.35	1.10–1.64	0.002	23,106	46.6	43,521	79.7	0.58	0.57–0.59	<0.001
2007	511,784	592,102	138	27.0	150	25.3	1.06	0.83–1.35	0.2	35,017	68.4	65,670	110.9	0.62	0.61–0.62	<0.001
2008	506,694	603,934	179	35.3	139	23.0	1.53	1.22–1.93	<0.001	53,428	105.4	99,637	165.0	0.64	0.63–0.64	<0.001
2009	501,142	615,333	157	31.3	108	17.6	1.78	1.39–2.30	<0.001	63,445	126.6	120,259	195.4	0.65	0.64–0.65	<0.001
2010	497,477	626,112	138	27.7	115	18.4	1.51	1.17–1.95	<0.001	65,343	131.3	128,330	205.0	0.64	0.63–0.65	<0.001
2011	494,379	635,183	97	19.6	105	16.5	1.19	0.89–1.58	0.1	80,277	162.4	161,494	254.2	0.64	0.63–0.64	<0.001
2012	491,862	642,927	111	22.6	95	14.8	1.53	1.15–2.03	0.001	108,134	219.8	217,413	338.1	0.65	0.64–0.65	<0.001
2013	489,469	649,234	98	20.0	90	13.7	1.44	1.07–1.94	0.006	138,174	282.3	272,753	420.1	0.67	0.67–0.68	<0.001

In the sensitivity analysis, there was a very good correlation between number of HIV-infected adults > = 15 years of age on ART and decreasing iNTS in our data (r = 0.93, p < .001) compared with the unpublished data from NDoH [18].

## Discussion

There is a high incidence of invasive salmonellosis in Africa, due to several important predisposing factors, including malaria, malnutrition and HIV infection [11,19]. In South Africa, the major contributing factor to invasive salmonellosis is HIV infection [20]: the introduction of ART is thus critical to preventing iNTS infections. We examined the incidence of iNTS in Gauteng Province, which has a predominantly urbanised population, and documented a significant decrease in the incidence of iNTS cases in a period of increased ART utilization which followed the introduction of ART in 2004.

A decrease in iNTS incidence has been described in other countries in Africa where malaria is endemic [14–16]. Malaria is an uncommon disease in South Africa, and Gauteng Province is malaria-free; therefore, we conclude that the decrease in iNTS incidence in South Africa is not due to malaria control efforts but due to the introduction of ART. In South Africa, almost all HIV-infected persons obtain ART through government HIV clinics. Our novel method for estimating the number of patients using ART takes advantage of the widely implemented HIV management protocol in South Africa that HIV-infected patients on ART should have viral load testing done at least annually to monitor their response to antiretrovirals [1]. Therefore, our study utilized data collected in a province where the population has good access to healthcare, and included almost comprehensive HIV viral load data from HIV-infected persons in this province. Other studies examining the association of tuberculosis and HIV in South Africa [8] utilised the less complete ART use estimates from ASSA which are based on prospective statistical modelling [3]. In our sensitivity analysis, we showed that our estimate of ART use, based on HIV viral load data, correlated well with the incomplete ASSA ART use estimates. However, because of the more robust data on viral load measurement tests used for our ART use estimates, we believe our estimate on ART is a better indication of the true number of patients accessing ART.

Tanser *et al* showed a decline in HIV acquisition in HIV-discordant couples in South Africa, supporting the importance of the national antiretroviral program in controlling HIV [21]. We demonstrate the additional significant impact of ART on prevention of iNTS infection. Although we only examined data from a single province, ART use has been implemented in South Africa and these findings can likely be extrapolated to other provinces.

We elected to separate children under five years from those aged 5–14 years for two reasons: firstly, as has previously been shown, children in the former age group are predisposed to a high mortality due to diarrheal diseases [22] and may thus have different predisposing factors for iNTS, including malnutrition. While this study did not expressly examine the role of malnutrition in children in association with iNTS, we have investigated this elsewhere: in South Africa, malnutrition does not contribute significantly to mortality due to iNTS infection, although HIV-infected children are more likely to be malnourished than HIV-uninfected children (P = 0.02) (Keddy et al, submitted). Secondly, there are differences in the HIV rates and clinical presentation between these two age groups; HIV-infected children living beyond five years were an unusual event prior to the introduction of perinatal ART and the ART roll-out.

Our data showed a significant correlation between the declining incidence of *Salmonella* Typhimurium and increasing incidence of patients accessing ART, suggesting this serotype specifically may act as an indicator pathogen for the response to ART in South Africa. More specifically, this decrease was observed across individual age groups and most notably in patients aged 25 to 49 years, who bear the highest burden of HIV infection in South Africa. An excessive burden of *Salmonella* Typhimurium, representing 85% of iNTS isolates, associated with multidrug resistance, has been described from other African studies and has been partly associated with HIV status [23]. We have previously described the predominance of *Salmonella* Typhimurium ST313, associated with iNTS meningitis in HIV- infected patients [12]: we suspect this particular pathogen, which emerged in Africa with the HIV epidemic [9], was equally responsible for much of the invasive disease we identified here.

The incidence of invasive disease due to *Salmonella* Enteritidis has increased in South Africa. This was observed across all age groups over the time period, despite the ART roll-out, and is not easily explainable. We would have expected that all invasive *Salmonella*, irrespective of serotype, would have decreased in association with ART usage. We suspect that this may have been due in part to ill-defined associations between food safety and food security: the mechanisms affecting the association between HIV infection, foodborne diseases, malnutrition and food insecurity globally have been described and may be at play here [24] The economic outlook of South Africa decreased dramatically over the time period of this study, with the gross domestic product (GDP) growth rate halving during the period (http://www.africaneconomicoutlook.org/en/statistics/table-2-real-gdp-growth-rates-2003-2013/), although the population increased by approximately 50% (www.statssa.gov.za). This may have affected normal practices in the preparation of safe foods within households. [24]. In addition, new evidence suggests that some strains of *Salmonella* Enteritidis, similar to *Salmonella* Typhimurium ST313, may have become adapted in Africa to human-to-human transmission [25]. Additionally, it is possible that due to the ubiquity of *Salmonella* Typhimurium, with well-described anthroponotic transmission of this pathogen [26], in the earlier years of the study that population immunity may have affected the decreasing incidence to this serotype, but as population immunity due to *Salmonella* Enteritidis was less common, the population may have been more at risk for infection and hence invasive disease, resulting in increased incidence rates. It is also possible that the increased incidence of *Salmonella* Enteritidis may reflect an increase in prevalence of *Salmonella* Enteritidis in the food supply, most likely in poultry. Interestingly, Muthumbi *et al* conversely found *Salmonella* Enteritidis was replaced by *Salmonella* Typhimurium over the comparable time period [16], possibly for similar reasons. *Salmonella* Typhimurium appears primarily associated with nosocomial transmission in South Africa [27]. As ever fewer HIV-infected patients are treated in hospital and HIV is managed as an outpatient disease, these nosocomial and anthroponotic routes will become less important and the traditional routes of transmission of *Salmonella*, including as a foodborne pathogen will become more important. We suspect that as HIV transmission is controlled through various management programs, the importance of other factors associated with iNTS in South Africa (Keddy *et al*, submitted), where malaria plays an insignificant role, will become more apparent: in the future, we may see disease trends comparable with those of industrialised countries in patients presenting with iNTS [11].

It is concerning that adult men appeared to access ART at a slower rate than adult women, which may be translating into a greater risk of opportunistic iNTS. Previously we showed approximately 90% of iNTS cases in adults are HIV-associated (Keddy *et al*, submitted) and assumed that this would impact on the rates of HIV-infected men versus women presenting with iNTS from 2003 to 2013. In 2003 and 2004, when ART was introduced, iNTS incidence rates per 100,000 HIV-infected adult men were less than those in adult women, but this reversed in 2005, and has remained so through to 2013, as ART became more accessible to the Gauteng population. Excess HIV-associated mortality in adult men, in association with a delayed ART access has previously been described in South Africa [17]: adult men may benefit from targeted interventions for HIV management programs.

This study has limitations. Firstly, although we attempted to comprehensively record Gauteng iNTS cases during the study period, some may have been were missed by our surveillance system. However, due to large numbers of patients with iNTS and extensive ART data from 2004 to 2013, any potential effect of missed cases on our analyses would be minimal. Secondly, some HIV clinics possibly were not following HIV-management guidelines in obtaining HIV viral load estimates for patients on ART, resulting in our underestimating the number of HIV-infected persons on ART; we judge this to have limited effect on our findings since such underestimations are more likely to have occurred in more recent years, when greater numbers of HIV-infected persons were on ART [4]. The most important limitation of this study is the ecological nature of the study design. However, the strong scientific evidence that ART prevents opportunist infections among HIV-infected persons clearly supports the plausibility of our finding that increased use of ART among HIV-infected persons is associated with a decreased incidence of iNTS infections, and our methodology was validated by our sensitivity analysis based on unpublished NDoH data.

In conclusion, we showed iNTS decreased dramatically in Gauteng Province, South Africa, particularly iNTS infections caused by *Salmonella* Typhimurium, during a period of increased ART utilization. Continual monitoring of iNTS and *Salmonella* Typhimurium in particular, may act as an early alert to further successes or potential failures in the HIV treatment program. Adult men in Gauteng Province may not be accessing ART programs optimally and should be the focus of targeted interventions. We also noted a concerning increase in invasive *Salmonella* Enteritidis cases, for poorly understood reasons, needing further elucidation.

### Ethics

Ethical approval for this study was granted by the Human Research Ethics Committee of the University of the Witwatersrand (M110601).

### Disclaimer

The findings and conclusions in this report are those of the authors and do not necessarily represent the official position of the US Centers for Disease Control and Prevention (CDC) or the National Institute for Communicable Diseases (NICD).

## Supporting information

S1 Table Population by age group (www.statssa.gov.za) per year, HIV-infected population (and prevalence per100,000 population) [3] and number of persons (and incidence per 100,000 population) accessing antiretroviral therapy (ART) per year based on viral load testing extracted from the Central Data Warehouse, Gauteng Province, South Africa, 2004–2013. (DOCX)

S2 Table Test for trend of incidence of invasive nontyphoidal *Salmonella* (all serotypes, *Salmonella* Typhimurium, and *Salmonella* Enteritidis) per 100,000 population per year, by age group, in Gauteng Province, South Africa. 2003–2013.
(DOCX)

S3 Table Incidence of invasive nontyphoidal *Salmonella* per 100,000 population per year by age group Gauteng Province, South Africa, 2004–2013. (DOCX)

S4 Table Incidence of invasive *Salmonella* Typhimurium per 100,000 population per year by age group, Gauteng Province, South Africa, 2004–2013. (DOCX)

S5 Table Incidence of invasive *Salmonella* Enteritidis per 100,000 population per year by age group, Gauteng Province, South Africa, 2004–2013. (DOCX)

S1 Dataset Invasive nontyphoidal *Salmonella*, Gauteng Province 2003–2013. (XLSX)
