# Supplementary material for: An association between decreasing incidence of invasive non-typhoidal salmonellosis and increased use of antiretroviral therapy, Gauteng Province, South Africa, 2003–2013
Source: PLoS One. 2017 Mar 6;12(3):e0173091. doi: 10.1371/journal.pone.0173091 (PMC5338796; doi:10.1371/journal.pone.0173091)
Supplement: S1 Table — (DOCX) [file pone.0173091.s001.docx]

S1 Table. Population by age group ([www.statssa.gov.za](http://www.statssa.gov.za)) per year, HIV-infected population (and prevalence per100,000 population) [3] and number of persons (and incidence per 100,000 population) accessing antiretroviral therapy (ART) per year based on viral load testing extracted from the Central Data Warehouse, Gauteng Province, South Africa, 2004 -2013.

| Year | <5 years | | | | | | | | 5 - 14 years | | | | | | | | 15 – 24 years | | | | | | | | |
| --- | --- | --- | --- | --- | --- | --- | --- | --- | --- | --- | --- | --- | --- | --- | --- | --- | --- | --- | --- | --- | --- | --- | --- | --- | --- |
|  | Population | HIV infected population  (prevalence) | | | | Population accessing ART  (incidence) | | | Population | | HIV infected population  (prevalence) | | | Population accessing ART  (incidence) | | | Population | | HIV infected population  (prevalence) | | | Population accessing ART  (incidence) | | | |
| 2003 | 978,638 | 36,016 | | (3,680.3) | | - | | - | 1,660,838 | | 7,973 | | (480.1) | - | | - | 1,849,565 | | 154,111 | | (8,332.3) | - | | - | |
| 2004 | 980,499 | 38,882 | | (3,965.5) | | 606 | | (61.8) | 1,709,956 | | 11,450 | | (669.6) | 649 | | (38.0) | 1,853,453 | | 146,135 | | (7,884.5 | 392 | | (21.1) | |
| 2005 | 977,015 | 40,552 | | (4,150.6) | | 2,669 | | (273.2) | 1,757,046 | | 15,686 | | (892.7) | 2,234 | | (127.1) | 1,868,051 | | 136,781 | | (7,322.1) | 1,758 | | (94.1) | |
| 2006 | 971,544 | 41,665 | | (4,288.5) | | 5,413 | | (557.2) | 1,800,450 | | 20,622 | | (1,145.4) | 4,587 | | (254.8) | 1,887,926 | | 127,827 | | (6,770.8) | 4,065 | | (215.3) | |
| 2007 | 961,731 | 44,081 | | (4,583.5) | | 7,229 | | (751.7) | 1,852,191 | | 27,338 | | (1,476.0) | 6,823 | | (368.4) | 1,911,049 | | 129,400 | | (6,771.2) | 5,691 | | (297.8) | |
| 2008 | 960,768 | 42,595 | | (4,433.4) | | 8,892 | | (925.5) | 1,892,937 | | 33,028 | | (1,744.8) | 9,580 | | (506.1) | 1,940,398 | | 122,200 | | (6,297.7) | 8,235 | | (424.4) | |
| 2009 | 971,248 | 40,967 | | (4,218.0) | | 9,302 | | (957.7) | 1,920,887 | | 39,209 | | (2,041.2) | 11,789 | | (613.7) | 1,972,974 | | 116,346 | | (5,897.0) | 8,845 | | (448.3) | |
| 2010 | 991,519 | 38,631 | | (3,896.1) | | 9,835 | | (991.9) | 1,937,694 | | 45,158 | | (2,330.5) | 13,550 | | (699.3) | 2,006,540 | | 111,300 | | (5,546.8) | 8,677 | | (432.4) | |
| 2011 | 1,013,711 | 35,804 | | (3,531.9) | | 8,628 | | (851.1) | 1,947,754 | | 50,491 | | (2,592.2) | 15,505 | | (796.0) | 2,039,462 | | 106,240 | | (5,209.2) | 11,439 | | (560.9) | |
| 2012 | 1,020,369 | 33,380 | | (3,271.40 | | 9,040 | | (886.0 | 1,970,982 | | 54,435 | | (2,761.8) | 18,290 | | (928.0) | 2,085,959 | | 102,004 | | (4,890.0) | 15,529 | | (744.5) | |
| 2013 | 1,025,336 | 31,185 | | (3,041.4) | | 9,049 | | (882.5) | 1,985,733 | | 57,132 | | (2,877.1) | 21,810 | | (1,098.3) | 2,134,067 | | 99,061 | | (4,641.9) | 19,883 | | (931.7) | |
| Year | 25 – 49 years | | | | | | | | | | | ≥50 years | | | | | | | | | | | | |  |
|  | Population | | HIV infected population  (prevalence) | | | | Population accessing ART  (incidence) | | | | | Population | | | HIV infected population  (prevalence) | | | | | Population accessing ART  (incidence) | | | | |  |
| 2003 | 4,445,156 | | 739,815 | | (16,643.2) | | - | | | - | | 1,339,249 | | | 39,093 | | | (2,919.0) | | - | | | - | |  |
| 2004 | 4,552,587 | | 789,410 | | (17,339.8) | | 5,396 | | | (118.5) | | 1,404,237 | | | 46,577 | | | (3,316.9) | | 474 | | | (33.8) | |  |
| 2005 | 4,653,273 | | 826,151 | | (17,754.2) | | 24,231 | | | (520.7) | | 1,475,210 | | | 53,999 | | | (3,660.4) | | 2,488 | | | (168.7) | |  |
| 2006 | 4,752,778 | | 852,415 | | (17,935.1) | | 56,850 | | | (1,196.1) | | 1,552,003 | | | 61,333 | | | (3,951.8) | | 6,340 | | | (408.5) | |  |
| 2007 | 4,843,434 | | 902,924 | | (18,642.2) | | 85,640 | | | (1,768.2) | | 1,633,885 | | | 71,562 | | | (4,379.9) | | 10,187 | | | (623.5) | |  |
| 2008 | 4,932,900 | | 909,624 | | (18,439.9) | | 128,965 | | | (2,614.4) | | 1,718,706 | | | 78,804 | | | (4,585.1) | | 16,768 | | | (975.6) | |  |
| 2009 | 5,023,297 | | 913,502 | | (18,185.3) | | 154,129 | | | (3,068.3) | | 1,805,526 | | | 86,628 | | | (4,797.9) | | 21,670 | | | (1,200.2) | |  |
| 2010 | 5,116,721 | | 916,937 | | (17,920.4) | | 161,513 | | | (3,156.6) | | 1,893,586 | | | 95,353 | | | (5,035.6) | | 24,610 | | | (1,299.7) | |  |
| 2011 | 5,217,804 | | 918,654 | | (17,606.1) | | 199,613 | | | (3,825.6) | | 1,983,575 | | | 104,668 | | | (5,276.8) | | 32,539 | | | (1,640.4) | |  |
| 2012 | 5,309,141 | | 918,380 | | (17,298.1) | | 265,938 | | | (5,009.1) | | 2,077,435 | | | 114,405 | | | (5,507.0) | | 45,953 | | | (2,212.0) | |  |
| 2013 | 5,410,194 | | 915,279 | | (16,917.7) | | 331,955 | | | (96,135.7) | | 2,173,107 | | | 124,363 | | | (5,722.8) | | 61,899 | | | (2,848.4) | |  |
