# Supplementary material for: An association between decreasing incidence of invasive non-typhoidal salmonellosis and increased use of antiretroviral therapy, Gauteng Province, South Africa, 2003–2013
Source: PLoS One. 2017 Mar 6;12(3):e0173091. doi: 10.1371/journal.pone.0173091 (PMC5338796; doi:10.1371/journal.pone.0173091)
Supplement: S2 Table — 2003–2013. (DOCX) [file pone.0173091.s002.docx]

S2 Table. Test for trend of incidence of invasive nontyphoidal *Salmonella* (all serotypes, *Salmonella* Typhimurium, and *Salmonella* Enteritidis) per 100,000 population per year, by age group, in Gauteng Province, South Africa. 2003 – 2013.

| Characteristic | IRR | (95% CI) | *P* |
| --- | --- | --- | --- |
| All nontyphoidal *Salmonella* | 0.91 | (0.90 – 0.92) | <0.001 |
| *Age group* |  |  |  |
| <5 years | 0.95 | (0.93 – 0.96) | <0.001 |
| 5 - 14 years | 0.95 | (0.91 – 0.99) | 0.03 |
| 15 - 24 years | 0.90 | (0.87 – 0.94) | <0.001 |
| 25 - 49 years | 0.89 | (0.88 – 0.90) | <0.001 |
| ≥50 years | 0.96 | (0.94 – 0.99) | 0.007 |
|  |  |  |  |
| *Salmonella* Typhimurium | 0.79 | (0.78 – 0.81) | <0.001 |
| *Age group* |  |  |  |
| <5 years | 0.85 | (0.82 – 0.87) | <0.001 |
| 5 - 14 years | 0.85 | (0.79 – 0.90) | <0.001 |
| 15 - 24 years | 0.78 | (0.72 – 0.82) | <0.001 |
| 25 - 49 years | 0.78 | (0.77 – 0.80) | <0.001 |
| ≥50 years | 0.82 | (0.78 – 0.85) | <0.001 |
|  |  |  |  |
| *Salmonella* Enteritidis | 1.14 | (1.12 – 1.17) | <0.001 |
| *Age group* |  |  |  |
| <5 years | 1.17 | (1.12 – 1.22) | <0.001 |
| 5 - 14 years | 1.22 | (1.10 – 1.36) | <0.001 |
| 15 - 24 years | 1.18 | (1.08 – 1.30) | 0.001 |
| 25 - 49 years | 1.11 | (1.08 – 1.14) | <0.001 |
| ≥50 years | 1.23 | (1.16 – 1.30) | <0.001 |

IRR, incidence rate ratio; CI, confidence interval
