# Supplementary material for: An association between decreasing incidence of invasive non-typhoidal salmonellosis and increased use of antiretroviral therapy, Gauteng Province, South Africa, 2003–2013
Source: PLoS One. 2017 Mar 6;12(3):e0173091. doi: 10.1371/journal.pone.0173091 (PMC5338796; doi:10.1371/journal.pone.0173091)
Supplement: S3 Table — (DOCX) [file pone.0173091.s003.docx]

S3 Table. Incidence of invasive nontyphoidal *Salmonella* per 100,000 population per year by age group Gauteng Province, South Africa, 2004 – 2013.

| Year | <5 years | | 5 - 14 years | | 15 – 24 years | | 25 – 49 years | | ≥50 years | |
| --- | --- | --- | --- | --- | --- | --- | --- | --- | --- | --- |
|  | Number of invasive *Salmonella* cases  (incidence) | | Number of invasive *Salmonella* cases  (incidence) | | Number of invasive *Salmonella* cases  (incidence) | | Number of invasive *Salmonella* cases  (incidence) | | Number of invasive *Salmonella* cases  (incidence) | |
| 2004 | 133 | (13.56) | 17 | (0.99) | 34 | (1.83) | 333 | (7.31) | 41 | (2.92) |
| 2005 | 119 | (12.18) | 16 | (0.91) | 30 | (1.61) | 298 | (6.40) | 51 | (3.46) |
| 2006 | 145 | (14.92) | 22 | (1.22) | 26 | (1.38) | 339 | (7.13) | 45 | (2.90) |
| 2007 | 132 | (13.73) | 23 | (1.24) | 24 | (1.26) | 225 | (4.65) | 42 | (2.57) |
| 2008 | 129 | (13.43) | 20 | (1.06) | 14 | (0.72) | 252 | (5.11) | 59 | (3.43) |
| 2009 | 103 | (10.60) | 15 | (0.78) | 28 | (1.42) | 192 | (3.82) | 49 | (2.71) |
| 2010 | 104 | (10.49) | 22 | (1.14) | 20 | (1.00) | 174 | (3.40) | 61 | (3.22) |
| 2011 | 77 | (7.60) | 13 | (0.67) | 11 | (0.54) | 146 | (2.80) | 49 | (2.47) |
| 2012 | 79 | (7.74) | 14 | (0.71) | 13 | (0.62) | 145 | (2.73) | 49 | (2.36) |
| 2013 | 65 | (6.34) | 9 | (0.45) | 15 | (0.70) | 128 | (2.37) | 45 | (2.07) |
