# Supplementary material for: An association between decreasing incidence of invasive non-typhoidal salmonellosis and increased use of antiretroviral therapy, Gauteng Province, South Africa, 2003–2013
Source: PLoS One. 2017 Mar 6;12(3):e0173091. doi: 10.1371/journal.pone.0173091 (PMC5338796; doi:10.1371/journal.pone.0173091)
Supplement: S4 Table — (DOCX) [file pone.0173091.s004.docx]

S4 Table. Incidence of invasive *Salmonella* Typhimurium per 100,000 population per year by age group, Gauteng Province, South Africa, 2004 – 2013.

| Year | <5 years | | 5 - 14 years | | 15 – 24 years | | 25 – 49 years | | ≥50 years | |
| --- | --- | --- | --- | --- | --- | --- | --- | --- | --- | --- |
|  | Number of invasive *Salmonella* Typhimurium cases  (incidence) | | Number of invasive *Salmonella* Typhimurium cases  (incidence) | | Number of invasive *Salmonella* Typhimurium cases  (incidence) | | Number of invasive *Salmonella* Typhimurium cases  (incidence) | | Number of invasive *Salmonella* Typhimurium cases  (incidence) | |
| 2004 | 84 | (8.57) | 11 | (0.64) | 22 | (1.19) | 248 | (5.45) | 30 | (2.14) |
| 2005 | 54 | (5.53) | 8 | (0.46) | 19 | (1.02) | 220 | (4.73) | 41 | (2.78) |
| 2006 | 84 | (8.65) | 16 | (0.89) | 18 | (0.95) | 263 | (5.53) | 29 | (1.87) |
| 2007 | 71 | (7.38) | 10 | (0.54) | 7 | (0.37) | 122 | (2.52) | 23 | (1.41) |
| 2008 | 53 | (5.52) | 12 | (0.63) | 9 | (0.46) | 145 | (2.94) | 31 | (1.80) |
| 2009 | 27 | (2.78) | 5 | (0.26) | 15 | (0.76) | 73 | (1.45) | 17 | (0.94) |
| 2010 | 32 | (3.23) | 10 | (0.52) | 6 | (0.30) | 68 | (1.33) | 21 | (1.11) |
| 2011 | 10 | (0.99) | 1 | (0.05) | 1 | (0.05) | 31 | (0.59) | 9 | (0.45) |
| 2012 | 12 | (1.18) | 3 | (0.15) | 1 | (0.05) | 19 | (0.36) | 6 | (0.29) |
| 2013 | 7 | (0.68) | 1 | (0.05) | 0 | (0.00) | 15 | (0.28) | 5 | (0.23) |
