# Supplementary material for: An association between decreasing incidence of invasive non-typhoidal salmonellosis and increased use of antiretroviral therapy, Gauteng Province, South Africa, 2003–2013
Source: PLoS One. 2017 Mar 6;12(3):e0173091. doi: 10.1371/journal.pone.0173091 (PMC5338796; doi:10.1371/journal.pone.0173091)
Supplement: S5 Table — (DOCX) [file pone.0173091.s005.docx]

S5 Table. Incidence of invasive *Salmonella* Enteritidis per 100,000 population per year by age group, Gauteng Province, South Africa, 2004 – 2013.

| Year | <5 years | | 5 - 14 years | | 15 – 24 years | | 25 – 49 years | | ≥50 years | |
| --- | --- | --- | --- | --- | --- | --- | --- | --- | --- | --- |
|  | Number of invasive *Salmonella* Enteritidis cases  (incidence) | | Number of invasive *Salmonella* Enteritidis cases  (incidence) | | Number of invasive *Salmonella* Enteritidis cases  (incidence) | | Number of invasive *Salmonella* Enteritidis cases  (incidence) | | Number of invasive *Salmonella* Enteritidis cases  (incidence) | |
| 2004 | 4 | (0.41) | 4 | (0.12) | 4 | (0.22) | 43 | (0.94) | 3 | (0.21) |
| 2005 | 9 | (0.92) | 9 | (0.11) | 2 | (0.11) | 26 | (0.56) | 3 | (0.20) |
| 2006 | 13 | (1.34) | 13 | (0.17) | 1 | (0.05) | 33 | (0.69) | 7 | (0.45) |
| 2007 | 19 | (1.98) | 19 | (0.11) | 5 | (0.26) | 42 | (0.87) | 6 | (0.37) |
| 2008 | 22 | (2.29) | 22 | (0.16) | 1 | (0.05) | 53 | (1.07) | 15 | (0.87) |
| 2009 | 24 | (2.47) | 24 | (0.10) | 3 | (0.15) | 60 | (1.19) | 17 | (0.94) |
| 2010 | 27 | (2.72) | 27 | (0.36) | 7 | (0.35) | 69 | (1.35) | 21 | (1.11) |
| 2011 | 32 | (3.16) | 32 | (0.41) | 6 | (0.29) | 80 | (1.53) | 28 | (1.41) |
| 2012 | 38 | (3.72) | 38 | (0.36) | 8 | (0.38) | 96 | (1.81) | 32 | (1.54) |
| 2013 | 40 | (3.90) | 40 | (0.40) | 12 | (0.56) | 83 | (1.53) | 32 | (1.47) |
